# Molecular Mechanisms Underlying Sugarcane Response to Aluminum Stress by RNA-Seq

**DOI:** 10.3390/ijms21217934

**Published:** 2020-10-26

**Authors:** Thiago Mateus Rosa-Santos, Renan Gonçalves da Silva, Poornasree Kumar, Pratibha Kottapalli, Chiquito Crasto, Kameswara Rao Kottapalli, Suzelei Castro França, Sonia Marli Zingaretti

**Affiliations:** 1 Functional Genomics Lab, Biotechnology Department, University of Ribeirão Preto, Ribeirão Preto, SP 14096-900, Brazil; thiagomateusrp@gmail.com (T.M.R.-S.); sfranca@unaerp.br (S.C.F.); 2 School of Agricultural and Veterinarian Sciences, São Paulo State University (UNESP), Jaboticabal, SP 14884-900, Brazil; biotek_rere@hotmail.com; 3 Center for Biotechnology and Genomics, Texas Tech University, Lubbock, TX 79409, USA; poorna12k@gmail.com (P.K.); chiquito.crasto@ttu.edu (C.C.); 4 Hartwell Center, St. Jude Children’s Research Hospital, Memphis, TN 38105, USA; pratibhak108@gmail.com; 5 AUA College of Medicine, Jabberwock Rd., P.O. Box 1451, Osbourn, Antigua and Barbuda; kottapallisri@gmail.com

**Keywords:** Aluminum ions (Al^3+^), *Saccharum*, Detoxification, Auxin signaling

## Abstract

Some metals are beneficial to plants and contribute to critical physiological processes. Some metals, however, are not. The presence of aluminum ions (Al^3+^) can be very toxic, especially in acidic soils. Considerable parts of the world’s arable land are acidic in nature; mechanistically elucidating a plant’s response to aluminum stress is critical to mitigating this stress and improving the quality of plants. To identify the genes involved in sugarcane response to aluminum stress, we generated 372 million paired-end RNA sequencing reads from the roots of CTC-2 and RB855453, which are two contrasting cultivars. Data normalization resulted in 162,161 contigs (contiguous sequences) and 97,335 genes from a *de novo* transcriptome assembly (trinity genes). A total of 4858 and 1307 differently expressed genes (DEGs) for treatment versus control were identified for the CTC-2 and RB855453 cultivars, respectively. The DEGs were annotated into 34 functional categories. The majority of the genes were upregulated in the CTC-2 (tolerant cultivar) and downregulated in RB855453 (sensitive cultivar). Here, we present the first root transcriptome of sugarcane under aluminum stress. The results and conclusions of this study are a crucial launch pad for future genetic and genomic studies of sugarcane. The transcriptome analysis shows that sugarcane tolerance to aluminum may be explained by an efficient detoxification mechanism combined with lateral root formation and activation of redox enzymes. We also present a hypothetical model for aluminum tolerance in the CTC-2 cultivar.

## 1. Introduction

Sugarcane (*Saccharum officinarum* L.), as any other plant, is constantly exposed to abiotic factors such as water availability (or lack thereof), temperature, soil composition, and metal toxicity. All these factors are extremely important for maintaining plant development and production and increasing yield. Though heavy metals such as copper (Cu), iron (Fe), and zinc (Zn) are essential for the physiological and biochemical processes, the presence of nonessential metals like aluminum ions (Al^3+^) in the soil can be toxic for plants. At neutral pH, aluminum exists in the harmless aluminosilicate or oxide forms. Under acidic condition (pH < 5.5), Al is solubilized as Al^3+^ ions, which are phytotoxic [1]. This is an important limiting factor for sugarcane production in acidic soils. Thirty percent of the world’s arable land is acidic; 60% of acidic arable land is found in the tropical and subtropical regions [2]. In Brazil, 500 million acres of arable land are acidic [3]. Most sugarcane farming occurs in acidic soil.

Plants’ response to abiotic stress involves a complex network that includes physiological and biochemical changes. The primary symptom of metal toxicity is root growth inhibition. The root apex is the most sensitive part of the root. The effects of metal toxicity can be detected just a few hours after exposure [4]. In general, aluminum causes fast inhibition of the elongation of the root’s main axis and reduction of lateral root development. This causes roots to be short and fragile [5]. Aluminum toxicity has also been reported in close association with nucleic acids [6] and is responsible for the blockage of cell division [7]. The physiological and transcriptional responses to the presence of aluminum can be different for different species, varieties, and cultivars, as they are the result of the differential expression of a set of genes. Using biotechnology techniques, the identification of those genes in sugarcane can help breeders take steps to increase tolerance in existing cultivars. These genes can also be used as biomolecular markers for selection in breeding programs.

The first and largest sugarcane ESTs (expressed sequence tags) database, with 237,954 sequences derived from 26 libraries, was obtained from different tissues and cultivars of sugarcane generated by the SUCEST (Sugarcane EST) project [8,9]. A total of 61.5% of the assembled sequences found matches in GenBank. The remaining nonmatched sequences likely represent new genes or alleles. In the last few decades, ESTs and cDNA collections had been used for differential gene expression, gene discovery, and the identification of regulation patterns in organisms, including sugarcane. Many differentially expressed genes have been reported for different stress conditions using different techniques such as quantitative PCR, microarrays, and SAGE (serial analysis of gene expression). Differential gene expression for water stress in tolerant and sensitive sugarcane cultivars has been reported [10,11,12], as have been reports of hormonal response for sugarcane ripening using microarrays [13]. Genes and proteins, differentially expressed under salt stress, and detected by RT-qPCR, two-dimensional gel electrophoresis, and mass spectrometry, have also been reported for sugarcane [14,15].

Currently, RNA sequencing (RNA-seq), a transcriptomic technology that uses high-throughput sequencing, has been used to analyze tissue or cell cDNA libraries. Because this methodology typically provides an exhaustive view of an organism’s transcriptome, the large number of resulting reads (numbering in the millions) obtained identifies RNA-seq as an important tool for transcriptome analysis. Studies had been carried out using RNA-seq to identify genes that are differentially expressed in the leaves of sugarcane cultivars under water stress [16], at different stages of leaf development [17], with leaf abscission [18], or under biotic stress from the pathogen *Fusarium verticillioides* [19]. Most of these studies focused on the commercial varieties of sugarcane, developed for the production of sugar and/or ethanol. Although genes implicated in the plant response to aluminum stress are already known in *Arabidopsis* [20], maize [21,22], alfalfa [23], soybean [24], and wheat [25], to our knowledge, no results in sugarcane transcriptome under Al stress conditions have been published. Understanding the molecular mechanisms of sugarcane response to aluminum stress is of fundamental importance for the development of high-performance plants in acidic soil. In this study, we carried out the first global transcriptomic analysis of sugarcane roots under aluminum treatment using the Illumina RNA-seq method. The identification of aluminum-responsive genes will extend our knowledge of sugarcane response to aluminum stress at the transcriptional level.

## 2. Results

### 2.1. Identification of Sugarcane Genotypes with Contrasting Aluminum Stress Tolerance

After hydroponic screening of four genotypes at high Al concentrations, CTC-2 (tolerant aluminum stress, TAS) and RB855453 (sensitive aluminum stress, SAS) were chosen as tolerant and sensitive sugarcane cultivars, respectively [26]. At higher aluminum concentrations, CTC-2 showed a higher tolerance to aluminum with more accumulation of biomass in the roots and higher dry mass, root density, root area, and proline content compared to the RB855453 genotype [26].

### 2.2. Phytotoxic Effects of Aluminum Ions (Al^3+^) on the Growth of Sugarcane Roots

**Figure 1:**
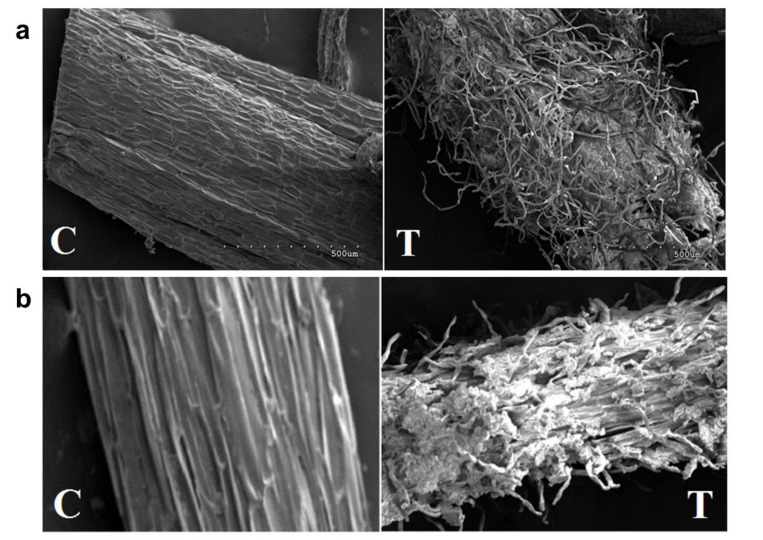
Root elongation zone photographed using electron microscope. (**a**) Roots of SAS; (**b**) roots of TAS. C = control (−Al); T = treatment (+Al). The images in (**a**) and (**b**) have the same scale and represent 500 µm.

The abundance of aluminum was determined in the root cap and elongation zone. An increase in the aluminum concentration was observed in the root cap for both cultivars, 3x in TAS and SAS compared to control plants (Table 1). For the elongation zone, on the other hand, the concentration of aluminum was quite different between the cultivars. The presence of aluminum in this region reveals that it has been translocated more efficiently by the tolerant cultivar. The increased concentration of aluminum in the root elongation zone could be responsible for the intense cellular disorganization observed in the roots (Figure 1a,b). However, the disruption in the cortex protoderm layers seems to happen in both cultivars.

### 2.3. De Novo Assembly of RNA-seq Reads for Generation of Reference Transcriptome

A transcriptome for sugarcane roots of 40.2 Gb was assembled using 372 million paired-end sequencing reads having read length of 108 bp from the roots of TAS and SAS genotypes, grown under aluminum stress. A Q-score (Phred quality score) of 38 indicates that the reads were of high quality. Reads were assembled into contigs using Trinity (*de novo* transcriptome assembly) software, with the default settings. Paired-end reads from both the genotypes were assembled with in silico normalization and the assembly statistics are summarized in Table S1. Normalization with 100 x coverage max per transcript resulted in 162,161 contigs and 97,335 trinity genes, with a Contig N50 of 1095. This indicates that 50% of the reads had contig lengths greater than or equal to 1095, and CG content of 49.68%. The length distribution of the Trinity-assembled contigs is presented in Supplementary Figure S1a. A total of 10,606 contigs were greater than two kilobases in length, potentially representing full-length cDNA sequences of sugarcane genes.

### 2.4. Differential Expression Analysis

The differentially expressed genes (DEGs) were identified between samples. The following statistically significant (twofold change, Benjamini and Hochberg-adjusted P-values (FDR) ≤ 0.05) DEGs were identified: 25,748 contigs between TAS treatment vs. control and 14,593 contigs between SAS treatment vs. control (Supplementary Figure S1b). After the read cut-off (contigs that had a sum of reads equal to and greater than 25 were used for analysis), 4858 differentially expressed genes were identified for the TAS treatment vs. control and 1307 contigs were differentially expressed in SAS treatment vs. control. While 464 contigs were common between the two genotypes, 4394 and 843 contigs were unique to TAS and SAS, respectively.

### 2.5. Functional Annotation of Consensus Sequence (Contigs)

**Figure 2:**
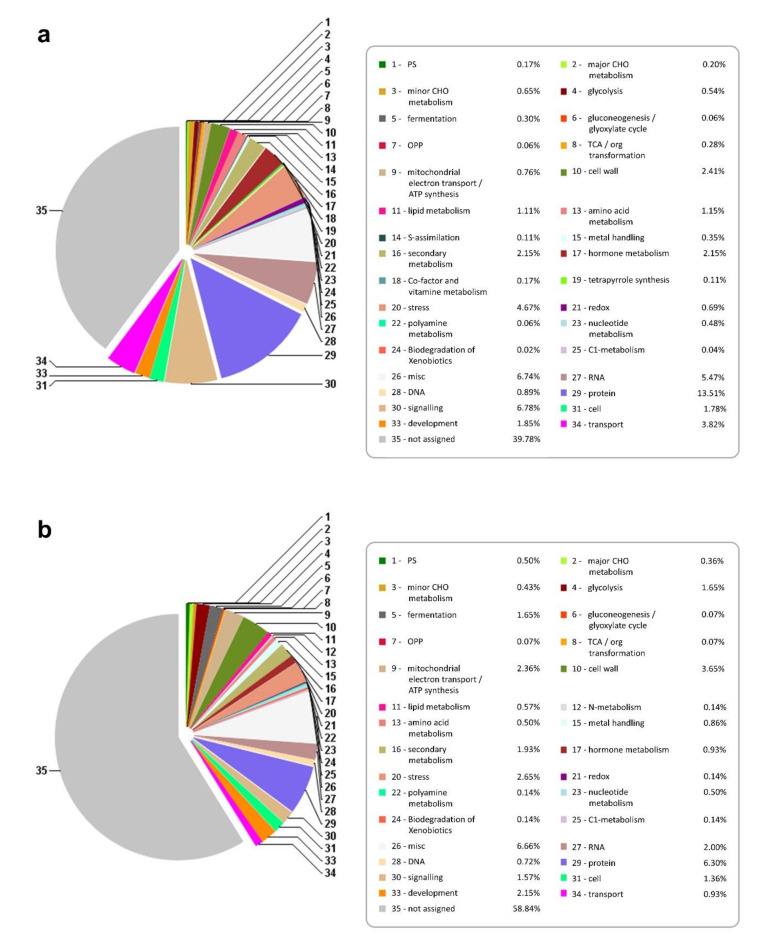
Mercator annotation of differentially expressed contigs in (**a**) TAS and (**b**) SAS.

**Figure 3:**
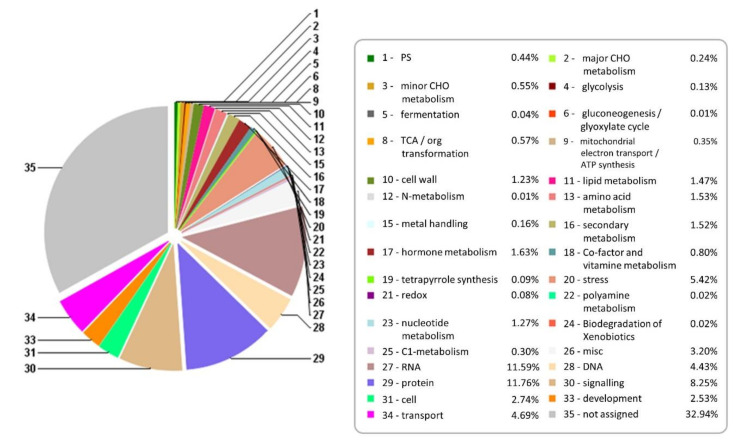
Mercator annotation of combined DEGs (differentially expressed genes in TAS and SAS).

The differentially expressed genes were annotated using the Mercator tool, which is used to annotate plant sequence data. It assigns functional annotations to protein and nucleotide sequences and assigns them into 34 functional MapMan bins. The results of Mercator annotation of 162,161 nonredundant contigs are presented in Figure 2. Over 50% of the contigs could be assigned a putative function. The contigs were binned into 34 different functional categories after annotation. As expected, the majority of annotated contigs had protein, RNA, signaling, transport, and stress functions (Figure 2). More than 8% of DEGS participate in signaling processes (Figure 3).

Although the functions identified as protein and RNA do not reflect the actual function of the genes, they represent a broad category of functions assigned under an abbreviated title. For example, the “protein” functional category had eight subcategories. It included functions like amino acid activation, protein synthesis, protein post-translational modification, protein degradation, and so forth. Similarly, the “RNA” category included RNA processing, transcription factors, regulation of transcription, and RNA binding. Annotated contigs presented as FASTA-formatted sequences from the sugarcane genotypes are available in a database maintained at the Center for Biotechnology and Genomics, Texas Tech University, USA.

### 2.6. Differential Gene Expression Analysis of Roots Exposed to Aluminum Stress

**Figure 4:**
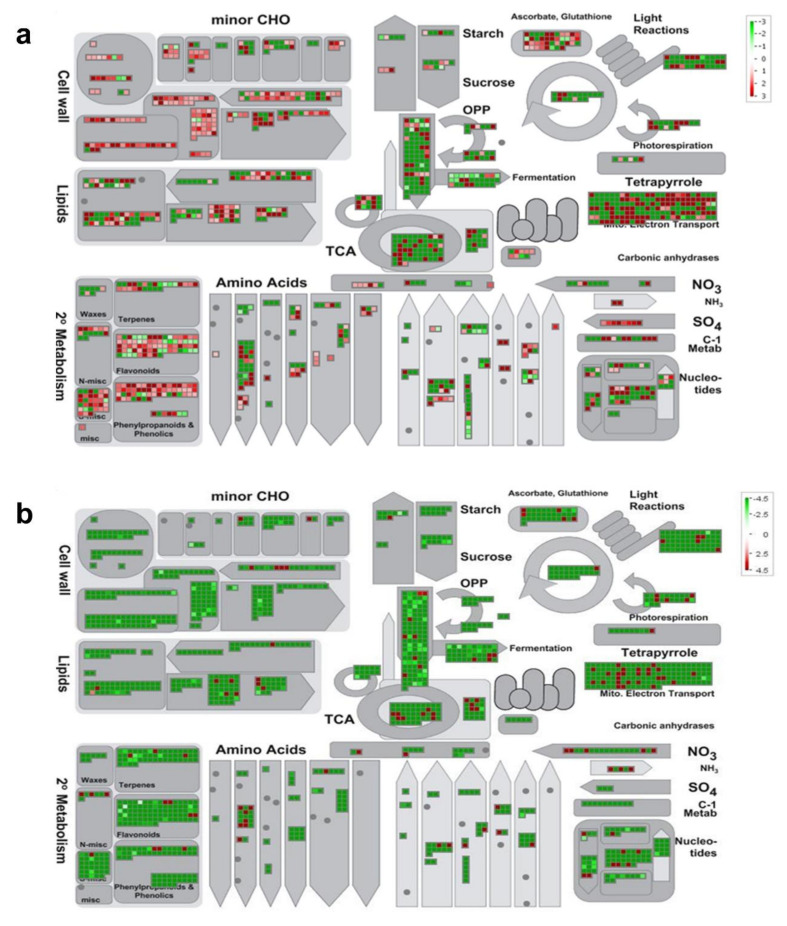
MapMan metabolic overview of aluminum-stress-responsive genes in sugarcane cultivars (**a**) TAS and (**b**) SAS. The analysis was performed using MapMan v.3.5.0. Small squares represent Log2 expression values of stress-responsive genes. The color key represents the RPKM (reads per kilobase million)—normalizes Log3-transformed counts. Red represents the upregulated and green the downregulated genes in response to aluminum stress.

Over 4800 contigs showed differential expression in TAS and 1307 in SAS cultivars under aluminum stress. The differentially expressed genes were mapped to biological pathways using MapMan software. DEGs are represented as colored squares. The red squares indicate the genes that are upregulated and the green squares indicate the genes that are downregulated. The members of the gene family are grouped together. An overview is presented for TAS and SAS (Figure 4a,b). The antagonist answer of those cultivars under aluminum stress response is very clear. The majority of genes are upregulated in TAS (Figure 4a) and downregulated in SAS (Figure 4b) especially in carbohydrate, cell wall, lipid, and secondary metabolism. This figure provides a global view of gene expression by mapping them onto functional pathways.

### 2.7. Mapping to Functional Pathways

**Figure 5:**
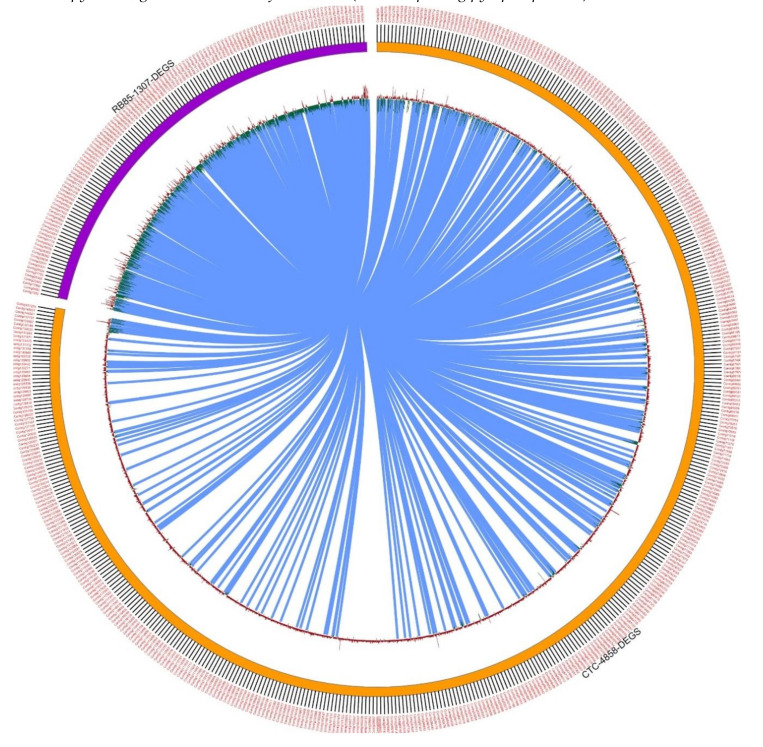
Circular visualization (Circos) plot showing differentially expressed genes showing shared biological processes for TAS and SAS. Two comparisons are plotted: CTC-2 control vs. treated with 4858 differentially expressed genes, and RB855453 control vs. treated with 1307 differentially expressed genes.

Differentially expressed contigs were mapped into metabolic pathways and other processes by MapMan v.3.6.0 [27]. The Mercator output file and the differentially expressed contigs with their Log_2_FC (fold-change) values were given as input for MapMan analysis. The same antagonist behavior can be seen in the circular visualization of DEGs of shared biological processes between the tolerant and the sensitive cultivars (Figure 5). Circos (http://circos.ca/) plots are a visualization tool that helps to represent networks in a circular fashion. The expression patterns of the differentially expressed contigs for TAS and SAS are represented by histograms. The upregulated contigs are represented by red bars and downregulated contigs are represented by green bars.

**Figure 6:**
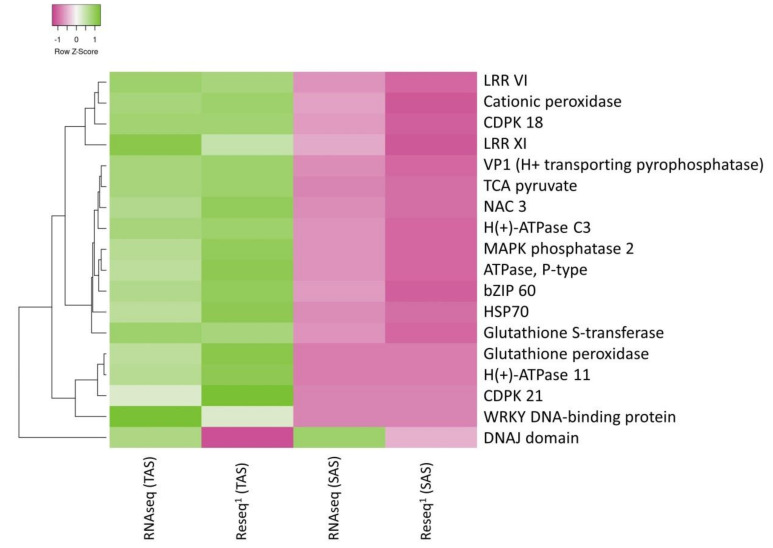
Hierarchical clustering heatmap based on the differential expression (row z-score) of selected genes. ^1^Reseq = Resequencing for validation.

Points that represent common contigs are graphically connected. From DEG analysis (Section 2.4), 4858 differentially expressed genes were identified for the TAS treatment vs. control (color-coded orange half of the pie) and 1307 contigs were differentially expressed in SAS treatment vs. control (color-coded purple half of the pie), while 464 contigs were common between the two genotypes (represented as the connecting links depicted as light blue lines). Using this tool, a large dataset can be visualized efficiently. We confirmed these results by resequencing other samples. Following resequencing, 11,164 genes were identified as differentially expressed when control and stress samples were compared for the tolerant cultivar and 4253 genes for the sensitive cultivar. The hierarchical clustering heatmap and Log_2_FC based on the differential expression for a sample of the DEG mRNA from both sequencing processes are presented in Figure 6 and Supplementary Table S2, respectively. Other genes validated in the resequencing are found in the Supplementary Table S3. The majority of the genes were upregulated in the tolerant cultivar and downregulated in the sensitive as for *TCA pyruvate*, *glutathione S-transferase*, *VP1* (*H+ transporting pyrophosphatase*), and *NAC 3*.

## 3. Discussion

### 3.1. Transcriptome Analysis

Transcriptomes of polyploidy plant species are often larger and more complex when compared to a diploid progenitor species. The complex architecture of transcriptomes comprising mRNA, small noncoding RNA, and long noncoding RNA necessitates a deep sequencing effort to understand the transcriptome of polyploidy sugarcane. Here, we have reported a first sugarcane transcriptome following aluminum stress. We generated 372 million paired-end sequencing reads of 108 bp length from roots of TAS and SAS genotypes that correspond to 40.2 Gb of sequence data. Assuming the sugarcane nuclear genome size as 10 Gb [28] and a transcriptome of 1.0 Gb (max 10% of genome as reported in humans), we have achieved approximately 40X coverage of the genome.

We assigned biological function to 5700 contigs: these included 4394 unique DEGs from TAS and 843 unique DEGs from SAS. These enabled us to assess the role of different cultivars. The abiotic stress caused by high concentrations of Al^3+^ is clearly different in treated and control plants. The number of upregulated genes is higher in the tolerant cultivar. By using contrasting cultivars, it was possible to narrow the search to genes really involved in the plant response to aluminum toxicity. The majority of identified DEGs common to both cultivars were distributed in the most abundant biological process: protein 11.76%, RNA 11.59%, signaling 8.25%, and transport 4.69% (Figure 3). Although Al, Cu, and other metals are important for normal plant growth, in high concentrations they will inhibit growth and increase reduction of lateral roots development [4].

### 3.2. Detoxification

Several genes related to detoxification and transport process, such as *zinc-induced facilitator-like 1* (*ZIFL1*), *TDT* (*tonoplast dicarboxylate transporter*), *metallothionein-like protein 1* (*MT1*), *ATPases, HIPP27*, *HIPP39* (*heavy metal-associated isoprenylated plant protein*), transcription factors: *ethylene-responsive element-binding factor* (*ERF014*), *dehydration-responsive element-binding protein, auxin response factor* (*ARF*) (role in lateral root formation), were identified and are essential for plant development under stress. An increase of Al concentration in the root cap and elongation zone was observed for both cultivars under Al^3+^ stress (Table 1), but the lower values observed in the elongation zone may be a result of changes in the metabolic responses of sugarcane plants altering the aluminum bioavailability. For the TAS cultivar, we observed root cellular disorganization and an increase in aluminum accumulation in the root cap and root elongation region. For the SAS cultivar, however, the level of aluminum in the elongation zone showed an enormous increase (800%) compared to those in SAS control plants and also similar cellular disorganization when compared to the TAS cultivar (Table 1 and Figure 1).

The availability of aluminum in plants can be associated with the structural effects on the root system, through positive or negative cell wall genes regulation (Figure 4) and consequent activation of mechanisms to minimize the Al stress effects on the plant. From the gene expression data, we observe that cell wall degradation enzymes such as endoglucanase-7, xyloglucan endotransglycosylase, pectate lyase, and polygalacturonase are upregulated in TAS and downregulated in SAS.

The aluminum effects on the root system can be perceived just a few hours after plants have been exposed [29]; the root cap is the main site of toxicity [30]. The primary effects reported in plants under aluminum stress are changes in the composition and properties of cell walls [31] as well as physiological, morphological, and anatomical modifications [32]. These effects observed in sugarcane roots (Table 1 and Figure 1), reported for corn [33] and wheat [31], are indicative of the different responses from TAS and SAS under aluminum stress conditions.

One of the immediate responses in this situation may be the detoxification of excess aluminum. Metallothioneins play an important role in heavy metal detoxification and homeostasis. These are metal-binding ligands that help in chelation and sequestration of heavy metal ions [34]. Here we observed that *MT1* is upregulated in the TAS cultivar and downregulated in the SAS cultivar. The *TCA pyruvate* and *ATPase, P-type* genes were highly induced in TAS and downregulated in SAS; the *H (+)-ATPase 11* gene was also upregulated under stress by TAS, while it was repressed in SAS. 

**Figure 7:**
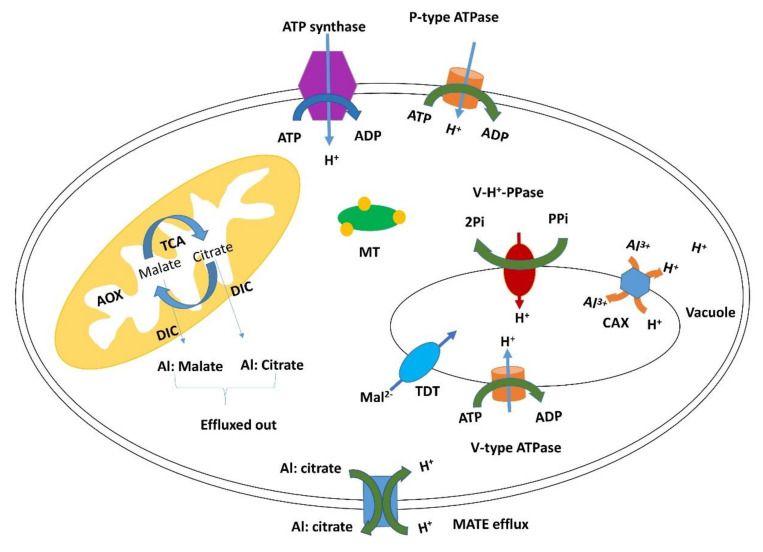
Detoxification mechanism used by TAS cultivar under aluminum stress.

The *TCA pyruvate* gene has been associated with metabolism and secretion of organic acids by being induced under aluminum stress in maize [35]. The induction of the *TCA pyruvate* gene promotes an internal aluminum detoxification mechanism, which increases the synthesis of malate and citrate organic acids that act as chelating agent (Figure 7). L-Malate and L-Citrate are secreted by special transporters such as MATE efflux proteins that had been identified in soybean under aluminum stress [36]. Those organic acid derivatives are especially important in the root cap [35]. They protect the roots by limiting Al access to cellular walls and prevent cellular disorganization [37]. ATPase P-type activities have been reported as induced by aluminum in an Al-resistant cultivar of wheat. This suggests that the induction of the enzyme could be an adaptive trait involved in Al resistance [38]. The differential expression pattern of *TCA pyruvate*, *ATPase (P-type)*, *H (+)-ATPase 11*, and MATE efflux protein transporters (Figure 6 and Supplementary Table S2) observed in this study can explain the efficient detoxification mechanism used by the TAS cultivar under aluminum stress (Figure 7).

### 3.3. Signaling

**Figure 8:**
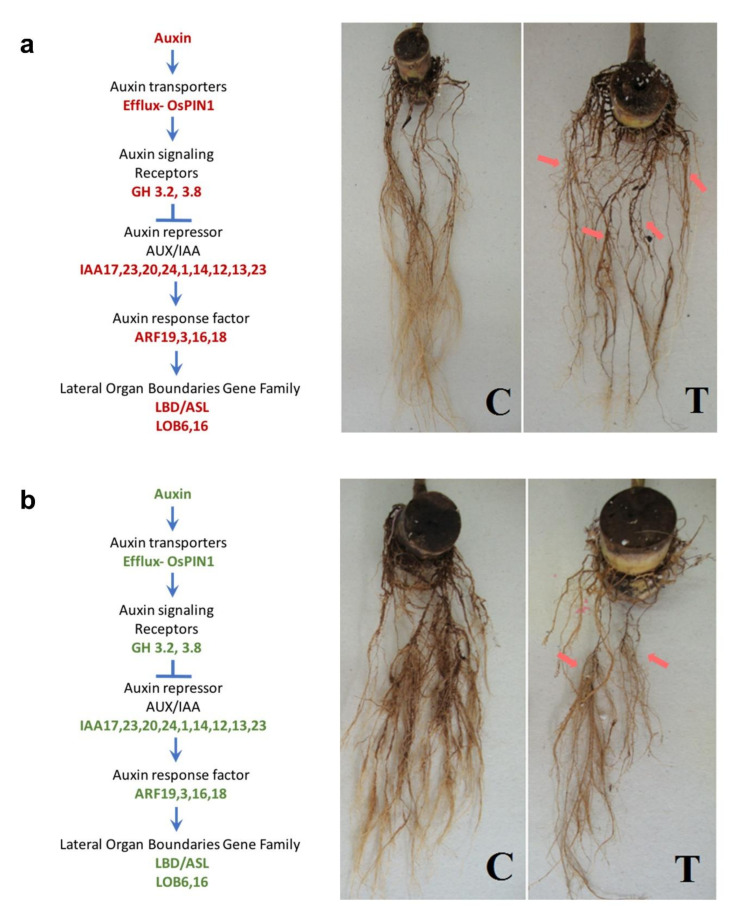
Auxin signaling pathway in sugarcane roots. Auxin transporters: OsPIN1—PIN-Formed auxin carrier component, GH3—indole-3-acetic acid amino synthetase, ARF—auxin response factor, LBD/ASL—lateral organ boundaries domain/asymmetric leaves2-like, LOB—lateral organ boundaries. The red and green colors indicate the upregulation and downregulation of the gene, respectively. The red arrows indicate the increased lateral root formation. (**a**) TAS—tolerant to aluminum stress, (**b**) SAS—sensitive to aluminum stress. C = control; T = treatment.

Auxin is essential for lateral root formation [39] and needs to be translocated to the lower part of the root. *Auxin efflux carrier components* (*PIN*) are auxin efflux proteins located in the subcellular organelles that are involved in polar transport of auxin [40]. The induction of transcription factors such as *PIN1*, *Aux/IAA*, and *ARF* will lead to an increase in the lateral root formation by the activation of the lateral organ boundaries domain/asymmetric leaves2-like genes *(LBD/ASL)* (Figure 8a). Additional lateral roots produced under aluminum stress play essential roles in the water and nutrient uptake required to sustain proper plant growth and development [41]. Our results showed that the transcriptional factors *PIN, Aux/IAA*, and *ARF* were induced in TAS and repressed in SAS, leading to an increase in lateral root formation on TAS and inhibiting it on SAS (Figure 8a,b). The inhibition of auxin flux was also reported in maize roots under aluminum stress [42]. Their results suggested an involvement of auxin transport inhibition in the expression of Al toxicity. The auxin response is also regulated by *auxin response factors* (*ARFs*) that can either activate or repress its expression. The *ARFs* also control the expression of *GH3* genes (*GH3.3*, *GH3.5*, and *GH3.6*), and their activity controls the initiation of adventitious roots (ARs) via modulation of auxin and jasmonic acid signaling routes in the *Arabidopsis* [43]. The degradation of *Aux/IAA* is required for the functioning of *ARF* [44]. In this study, *ARF19*, *ARF3*, *ARF18*, and *ARF6* were upregulated. 

### 3.4. ROS Protection

Heavy metal stress induces changes in free radical product and also in the antioxidant profiles. The peroxidation of membrane lipids is one of the common consequences of aluminum stress. This induces the expression of several genes that code for enzymes such as peroxidases, glutathione S-transferase, which play an important role in the antioxidant defense mechanism [45,46]. We have observed upregulation of peroxidases such as *L-ascorbate peroxidase 3*, *Glutathione peroxidase 2*, and *Glutathione S-transferases* in the TAS cultivar (tolerant) (Figure 6 and Supplementary Table S2), and downregulation of these genes in the SAS (sensitive) under aluminum stress. Studies have shown accumulation of ascorbate peroxidases during heavy metal stress. Increased ascorbate peroxidases have been reported during Al stress [47]. In *Arabidopsis*, the overexpression of *AtPrxs* gene, a peroxidase enzyme, increased the root growth and reduced the accumulation of Al and ROS in the roots [48]. Studies have also shown that anionic peroxidases activities occur in the cell wall, modulating and reducing the aluminum diffusion [49].

### 3.5. Signal Transduction 

**Figure 9:**
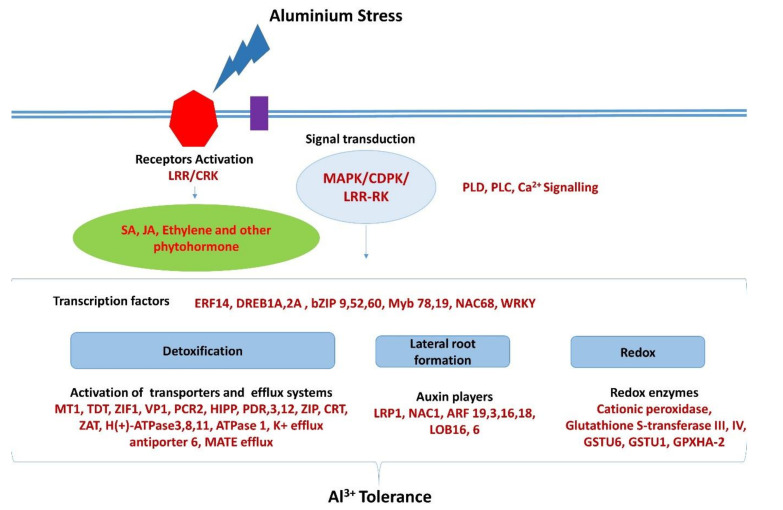
Hypothetical model for aluminum tolerance in TAS. Genes in red are upregulated.

The transcript data provided here point out that the tolerance of TAS may be explained by the perception of stress and the activation of transcription factors that will activate genes of three mechanisms of response: an efficient detoxification mechanism, lateral root formation, and activation of redox enzymes. The mechanism of efficient detoxification and auxin signaling activation leads to an increase in lateral roots formation, and the action of an efficient system of antioxidant enzymes. A hypothetical model for the aluminum tolerance in TAS is presented (Figure 9). 

During Al stress, the cell wall surface receptors such as *leucine-rich repeat* (*LRR*) and *cysteine-rich receptor-like protein kinase* (*CRK*) are upregulated in TAS and downregulated in SAS. We also have similar gene expression in other signaling proteins such as *mitogen-activated protein kinase* (*MAPK*), *calcium-dependent protein kinase* (*CDPK*), and others, all of which regulate the expression of various transcription factors. These in turn are responsible for several pathways for detoxification and protection from oxidative stress and also in lateral root formation conferring aluminum tolerance.

## 4. Materials and Methods

### 4.1. Sample Selection and Preparation of Collection

The sugarcane cultivars used in this study were kindly provided by Usina São Martinho (Usina São Martinho, Pradópolis, Brazil). Pregerminated plants, 60 days old, from the sugarcane (*Saccharum* spp) cultivars CTC-2, also tolerant to aluminum stress (TAS), and RB855453, also called sensitive to aluminum stress (SAS), were used in the present study. These genotypes were chosen as a contrast, based on previously published results [26]. The plants were grown using a hydroponic system in a greenhouse at 26 °C to 30 °C and with 8/16 h dark/light cycles. For 30 d, the plants were kept in 16 L containers filled with standard hydroponic solution [50] before cultivation for a seven-day period with the addition of either 0.0 or 221 µmol Al^3+^ L-1 at pH 4.5. After seven days, roots were collected and immediately frozen in liquid nitrogen and stored at –80 °C for further use.

### 4.2. Aluminum Abundance in Root

For aluminum relative abundance determination, root sample segments (1 cm long) from root cap and root elongation area of TAS and SAS cultivars were analyzed using a scanning electron microscope (LEO 1430 VP, Zeiss, Cambridge, UK) coupled to an X-ray probe (X-EDS, IXRF systems, Houston, TX, USA).

### 4.3. RNA Extraction, Library Preparation, and Sequencing

Total RNA was isolated from lyophilized powdered root tissue samples using the Spectrum™ Plant Total RNA kit (Sigma-Aldrich, St. Louis, MO, USA). The samples consisted of three biological replicates (plants from each treatment). RNA quality and concentration were quantified using the Qubit® RNA assay on a Qubit® 2.0 fluorometer (Life Technologies, Carlsbad, CA, USA). TruSeq RNA sample preparation kit (Illumina, Inc, San Diego, CA, USA) protocol was used for library constructions. Library quality was checked on TapeStation 2200 (Agilent, Santa Clara, CA, USA) using the D1K tape for validating the insert size and purity.

Paired-end sequencing of 12 cDNA libraries was done using the Illumina HiSeqTM2500 v2 platform (HiSeq 2500, Illumina Inc., San Diego, CA, USA) for a read length 2 × 108 bp. 

### 4.4. De Novo Transcriptome Assembly and Gene Functional Annotation

The raw sequencing data were quality checked using FastQC software (V0.11.6) (https://www.bioinformatics.babraham.ac.uk/projects/fastqc/). All sequencing reads obtained from both cultivars were then assembled to generate a single *de novo* reference transcriptome using Trinity software (V2.3.1) [51]. The Trinity script was modified according to computing requirements and k-mer-based in silico normalization (maximum coverage) was set to 100. The sequencing reads were mapped on the *de novo* reference transcriptome using DNASTAR’s Lasergene Genomics Suite (DNASTAR, Inc., Madison, WI, USA) which includes QSeq application of ArrayStar software.

The expression level of each transcript was normalized by reads per kilobase of the target per million mapped reads (RPKM). Differentially expressed genes exhibiting twofold changes and Benjamini and Hochberg-adjusted P-values (FDR) ≤ 0.05 were selected for the study.

Annotations of DEGS were predicted using the Mercator tool (V3.6) (http://mapman.gabipd.org/web/guest/app/Mercator). The Mercator tool generates functional predictions by scanning a variety of reference databases (three BLAST-based, two RPSBLAST-based, and InterProScan) and subsequently evaluating and compiling the search results for each input gene to propose a functional Bin based on the manually curated binning of the reference database entries [52]. Pathway mapping was performed by Mapman software (V3.5.1) (http://mapman.gabipd.org) and comparative transcriptome analysis by Circos plot. We used the Heatmapper software (http://www.heatmapper.ca/) [53] to provide a hierarchical clustering heatmap based on the differential expression (row z-score) of selected genes.

### 4.5. Resequencing for Validation

Twelve cDNA libraries were generated from the RNA samples used for initial sequencing using TruSeq RNA sample preparation kit (Illumina, Inc, San Diego, CA, USA). Libraries were resequenced on NovaSeq 6000 platform (Illumina Inc., San Diego, CA, USA) with paired-end sequencing of 2 × 108 bp read length on an S1 flow cell. The reads were mapped to the earlier assembled de novo reference transcriptome from the original sequencing using DNASTAR’s Lasergene Genomics Suite which includes QSeq application of ArrayStar software. The differentially expressed transcripts were used for validating the sequencing results.

### 4.6. Statistical Analysis

The obtained data for relative abundance of Al (EDS quantification) were submitted to analysis of variance (ANOVA), and the differences between means were evaluated by Tukey’s tests (p ≤ 0.01) using the AgroEstat sotware (https://www.agroestat.com.br/).

### 4.7. Availability of Data

All the data generated or analyzed during this study are included in this article and in supplementary information files. The raw sequence data can be accessed in the NCBI with accession number PRJNA544532.

## 5. Conclusions

The identification of a large number of differentially regulated transcripts opens up opportunities for the development of molecular markers associated with aluminum tolerance in sugarcane breeding programs. In the present study, the transcriptome of sugarcane roots was analyzed using RNA-seq technology to explore the mechanism(s) involved in aluminum tolerance. Genes related to the auxin signaling, detoxification, ROS protection, and signal transduction were significantly involved in the response of sugarcane roots to aluminum. Our findings provide numerous genes that can be applied in further research to improve the aluminum tolerance of functional plants and will enhance research on aluminum-tolerant mechanisms.

## Figures and Tables

**Table 1:** Relative abundance of Al in root cap and root elongation region of TAS and SAS plants, detected by energy-dispersive spectroscopy (EDS).

Cultivars	Condition	Aluminum Quantification–by EDS*	
		Root Cap	Root Elongation
TAS	Control	0.46 ± 0.02	0.22 ± 0.02
	Treatment	1.76 ± 0.03 **	0.44 ± 0.02 **
SAS	Control	0.41 ± 0.02	0.05 ± 0.02
	Treatment	1.37 ± 0.03 **	0.45 ± 0.02 **

* Values represent the average of the relative abundances (%) for all collection sites ± standard errors. ** Indicates significant difference between control and treatment (Tukey’s tests, *p* ≤ 0.01).

## Supplementary Materials

Supplementary materials can be found at https://www.mdpi.com/1422-0067/21/21/7934/s1. **Table S1.** *De novo* assembly statistics of sugarcane root transcriptome. **Table S2.** Differentially expressed genes validated by resequencing. **Table S3.** TAS and SAS contigs identified in sequencing and resequencing. **Figure S1.** Trinity assembled reads distribution and DEGS comparisons between (a) TAS and (b) SAS.

## Abbreviations

ESTs	expressed sequence tags
SUCEST	sugarcane EST
SAGE	serial analysis of gene expression
TAS	tolerant to aluminum stress
SAS	sensitive to aluminum stress
X-EDS	energy-dispersive X-ray spectroscopy
C	control
T	treatment (+Al)
DEGs	differentially expressed genes
FC	fold-change
Reseq	Resequencing
NI	not identified
