# Supplementary material for: Molecular Mechanisms Underlying Sugarcane Response to Aluminum Stress by RNA-Seq
Source: Int J Mol Sci. 2020 Oct 26;21(21):7934. doi: 10.3390/ijms21217934 (PMC7672567; doi:10.3390/ijms21217934)
Supplement: Supplementary file 1 [file ijms-21-07934-s001.zip › SUPPLEMENTARY MATERIAL_Table and Figure.docx]

**SUPPLEMENTARY MATERIAL**

**Molecular mechanisms underlying sugarcane response to aluminum stress**

**by RNA-Seq**

Thiago Mateus Rosa-Santos^a^, Renan Gonçalves da Silva^b^, Poornasree Kumar^c^_,_ Pratibha Kottapalli^c^, Chiquito Crasto^c^, Kameswara Rao Kottapalli^c^, Suzelei Castro França^a^, Sonia Marli Zingaretti^a,b^

**TABLE AND FIGURE**

**Table S1.** *De novo* assembly statistics of sugarcane root transcriptome.

| Total trinity 'genes': | 97,335 |
| --- | --- |
| Total trinity transcripts: | 162,161 |
| Percent GC: | 49.68 |
| Contig N50: | 1,095 |

**Table S2.** Differentially expressed genes validated by re-sequencing.

| **Contigs Description** | **TAS*** | | **SAS*** | |
| --- | --- | --- | --- | --- |
|  | **RNAseq** | **Reseq^1^** | **RNAseq** | **Reseq^1^** |
| *HSP70* | 5.45 | 10.60 | -9.35 | -11.29 |
| *TCA pyruvate* | 9.12 | 10.70 | -9.38 | -11.18 |
| *Glutathione S-transferase* | 9.72 | 8.09 | -8.17 | -11.85 |
| *ATPase, P-type* | 5.56 | 10.00 | -7.11 | -10.20 |
| *bZIP 60* | 7.61 | 10.57 | -5.81 | -10.00 |
| *H(+)-ATPase 11* | 8.24 | 10.49 | N I | N I |
| *MAPK phosphatase 2* | 6.82 | 10.41 | -7.09 | -10.12 |
| *Cationic peroxidase* | 1.59 | 2.37 | -6.42 | -9.22 |
| *Glutathione peroxidase* | 7.67 | 10.63 | N I | N I |
| *CDPK 21* | 5.72 | 11.18 | N I | N I |
| *CDPK 18* | N I | N I | -8.56 | -10.64 |
| *LRR XI* | 6.30 | 1.14 | -6.95 | -11.62 |
| *LRR VI* | 1.78 | 1.41 | -8.04 | -10.15 |
| *WRKY DNA-binding protein* | 5.27 | 2.75 | N I | N I |
| *VP1 (H+ transporting pyrophosphatase)* | 9.53 | 11.03 | -8.18 | -11.17 |
| *DNAJ domain* | -7.37 | -10.38 | -7.08 | -9.34 |
| *H(+)-ATPase C3* | 9.40 | 10.81 | -6.66 | -10.60 |
| *NAC 3* | 7.89 | 10.78 | -8.10 | -10.87 |

*Log2 Fold Change; ^1^Reseq = Re-sequencing for validation; N I = not identified.

**Figure S1.** Trinity assembled reads distribution (A) and DEGS comparisons between TAS and SAS (B).

**(A)**


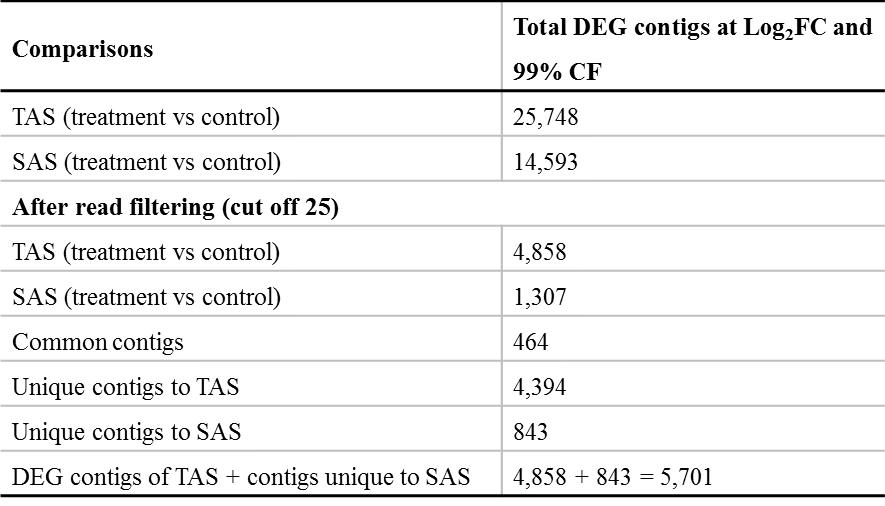


**(B)**
